# Experiences and perceptions of people with celiac disease, food allergies and food intolerance when dining out

**DOI:** 10.3389/fnut.2024.1321360

**Published:** 2024-02-02

**Authors:** Ximena Figueroa-Gómez, María Jesus Oliveras-López, Juan Manuel Rodríguez Silva, Marcelo Poyanco, Herminia López, Magdalena Araya

**Affiliations:** ^1^PhD Student of the Nutrition and Food Science Doctoral Program, Department of Nutrition and Bromatology, Faculty of Pharmacy, University of Granada, Granada, Spain; ^2^PhD Student of the Nutrition and Food Science Doctoral Program, Human Nutrition Unit, Institute of Nutrition and Food Technology (INTA), University of Chile, Santiago, Chile; ^3^Department of Molecular Biology and Biochemical Engineering, University Pablo de Olavide, Sevilla, Spain; ^4^Institute of Nutrition and Food Technology (INTA), University of Chile, Santiago, Chile; ^5^Faculty of Economic and Administrative Sciences, University of Valparaíso, Valparaíso, Chile; ^6^Department of Nutrition and Bromatology, Faculty of Pharmacy, University of Granada, Granada, Spain

**Keywords:** consumers, celiac disease, food allergies, food intolerances, food safety, food service

## Abstract

**Introduction:**

Eating out is a common practice in modern society. Celiac disease (CeD) and food allergy (FA) are among the most common conditions responsible for adverse reactions to food. Despite their different origins, both require treatment with restrictive diets (avoidance of gluten and/or specific allergens) and this results posing similar challenges when eating out. Our objective was to learn about the experiences/perceptions of consumers with CeD and FA when dining out, as well as the challenges they face in food service environments.

**Methods:**

An *ad hoc* questionnaire was used to record consumer perceptions, food service characteristics and resulting adverse reactions.

**Results:**

377 individuals living in Santiago, Chile, provided complete information and were analyzed (160 CeD, 105 FA). 301 participants (79.8%) declared eating out, 33.6% reported experiencing an adverse reaction at least once while eating out. 94.4% of the 377 participants believed that the serving staff had little or no knowledge about his/her condition. Consumers reporting symptoms as severe adverse reactions were more common among celiac than allergic patients (*p* < 0.001).

**Discussion:**

The study showed no significant differences based on consumer-related characteristics (*p*:NS). The consequences of eating out did not vary based on individual’s data, including diagnosis, age, frequency of eating out, adverse reactions experienced, or intensity. These findings suggest that the most important determinants of risk associated with eating out are characteristics of the food service, like availability of information, staff training, and establishment’s facilities like equipment available, exclusive utensils for customers with special dietary needs and kitchen and bathrooms organization.

## 1 Introduction

The incidence of adverse food reactions has increased in recent decades and is now a growing health problem (1, 2). Of these, celiac disease (CeD) and food allergy (FA) are among the most common. It is currently estimated that ~1% of the population suffers from CeD, although its prevalence is thought to be underestimated (3, 4). It is difficult to determine the frequency of FA; available data estimates that 32 million people in the United States (5) and 11–26 millions in Europe (1) are allergic to some food(s), with children developing FA more frequently (5–8%) than adults (1–2%) (6–8). Extrapolating these figures globally, some authors estimate that up to 1.6 billion people may be affected by FA worldwide (9).

### 1.1 Celiac disease

At present this is one of the most common autoimmune diseases, it develops when genetically susceptible individuals consume gluten containing foods, a protein found in wheat, barley, and rye, which results in a variety of gastrointestinal and extraintestinal symptoms (10). Symptoms vary greatly from person to person, from severe to minimal or no symptoms at all. The time it takes for symptoms to appear after gluten ingestion also varies, within a few hours to some days. Interestingly, some patients may remain asymptomatic despite severe damage to the small intestine (11). Up to date, the only effective treatment for CeD is elimination of gluten from the diet.

### 1.2 Non-celiac wheat sensitivity

This is a relatively newly described condition in which individuals develop intestinal and extraintestinal symptoms after eating wheat. Symptoms are clearly triggered by wheat ingestion, but the mechanisms by gluten it causes symptoms are unclear; recent reports describe that both typical genetic and autoimmune responses of CeD and the immunoglobulin E-mediated tests typical of wheat-FA are absent in non-celiac wheat/gluten sensitivity (12, 13). It is still uncertain which wheat proteins are responsible for inducing symptoms since there is evidence showing that not only gluten but also fructanes and amylase trypsin-inhibitors can activate immunity.

### 1.3 Food allergy

Food allergies are adverse immune reactions triggered by various foods, mainly milk, eggs, peanuts, tree nuts, shellfish, fish, wheat, and soy (14), which represent the most common allergens, although individuals can become sensitized to any food or ingredient. Symptoms of FA include gastrointestinal, skin, respiratory, neurological manifestations, among others (15). As for wheat allergy, symptoms range from mild to severe, is mostly mediated by IgE (with risk of life-threatening anaphylaxis) and triggered by proteins contained in wheat, not necessarily gluten (2, 16).

### 1.4 Food intolerance

One of the most challenging issues today is that some individuals clearly report symptoms after eating, but no clear diagnosis can be established. The term food intolerance is commonly used to describe this situation. The mechanisms responsible for inducing symptoms differ, like presence of toxins, enzymatic deficiencies, poor digestion of fermentable carbohydrates, etc. The most common condition in this chapter is lactose intolerance which is currently considered that it might be part of FODMAP (Fermentable Oligo-di-Monosaccharides and Polyols) sensitivity, but this latter is a wider set containing lactose intolerance. Caused by lactase (beta-D-galactosidase) deficiency, greater amounts of lactose remain in the intestinal lumen and reach the colon, where the intestinal microbiota metabolize it, producing gases and metabolites responsible for symptoms (17). Their severity vary from person to person, depending mainly on the degree of enzyme deficiency and the amount of food ingested. The typical clinical presentation includes colicky abdominal pain, bloating, flatulence and acidic stools; occasionally, some cases present decreased intestinal motility with constipation, partly related to methane production (17), although the evidence in favor of constipation induced by lactose intolerance is weak (18).

*Treatment with the Gluten-Free Diet* represent an increasing burden to healthcare systems, affected individuals and their families and caregivers (2, 17). Gluten related disorders are clinically different, and consequences of consuming the offending food also differ. While in wheat allergy symptoms develop within minutes to a few hours, in the other gluten-related conditions symptoms take longer to develop. Because gluten is able to induce autoimmune responses, any gluten ingestion potentially increases the risk of complications. To date, the only effective treatment for all these disorders is the “gluten-free diet” (19). All these patients follow gluten-free diet because the only products available in the market are the so-called “gluten-free products.” Though people with fructanes intolerance could eat “gluten-friendly” menu options, which are more available, celiac persons must eat ‘strict gluten-free’, avoiding all gluten cross-contamination.

In the last decades, modern life has changed eating habits and eating processed foods is a main trend in western societies. Availability of adequate information on the composition of processed foods is crucial to choose safe foods when shopping and eating. Labeling is increasingly regulated to ensure the safety of gluten-free and allergen-free products, but several situations in daily life remain uncertain. A relevant one relates to food services, restaurants, cafeterias, etc., common places where adverse reactions to food are triggered by hidden allergens/gluten and poor staff knowledge, among others (20).

Provision of written allergen information for pre-packaged foods is required by law in the United States (21) and Europe (22). The Codex Alimentarius defines gluten-free products as those whose gluten content is below a cutoff at 20 ppm (20 mg per kilogram of product) (23). But these regulations are not fully applied in many countries; moreover, gluten is widely used as ingredient/additive by the food industry, making difficult to properly comply with the gluten-free diet. The correct application of these regulations requires governmental commitment, informed consumers, and that food industries and restaurants/food services provide adequate information to customers and a staff trained on health and safety protocols able to accommodate special dietary needs.

Dining out should be a pleasant experience, allowing social interaction and the enjoyment of sharing food with family and friends. However, it becomes a threatening situation if the dietary requirements are not properly managed and provided (24). Little is known about celiac persons dining out (24, 25). Studies on FA show that affected persons feel lack information (26), anxiety and that their quality of life is deteriorated (27). COVID19 pandemic may have changed dietary habits due to food shortage and crowding (28), but its effects remain unclear. In this context, the objective of this study was to evaluate experiences and perceptions of people with dietary restrictions, mainly CeD and FA, when they go to eat out.

## 2 Materials and methods

This population-based study evaluated a non-probabilistic convenience sample of people living in Chile, mainly in Santiago (capital city).

### 2.1 Inclusion criteria

Women and men over 14 years of age who reported CD and/or FA and/or food intolerance were included. For children under 14 years of age with the same reported diagnoses, parents or guardians provided the necessary information for the study.

### 2.2 Exclusion criteria

Being under two years of age, breast feeding mothers, incomplete data provided in the questionnaire.

### 2.3 Questionnaire

An *ad-hoc* questionnaire was developed based on literature data and the authors’ own experience. It consisted of 32 questions, answered using Google’s survey management software (Google Forms). The questionnaire collected sociodemographic data, basic clinical characteristics, and frequency and habits of eating out, including questions on behavior, attitudes, perception of the level of knowledge about celiac disease and food allergy among foodservice staff and general feeling of safety when eating out. The questionnaire was validated by administering it to 37 apparently healthy adults and subsequently modified as necessary before starting the protocol. Some questions were multiple choice, others were answered yes/no or in a Likert scale. Anonymity and confidentiality were guaranteed. The study was conducted online between August and December 2021, and a total of 390 individuals completed the survey.

### 2.4 Operational procedure

The invitation to participate answering the online questionnaire was published on the websites of the University of Chile,^1^ Corporación de Apoyo al Celíaco (Coacel)^2^ and Fundación Creciendo con Alergias^3^, and disseminated through instant messaging and social networks (mainly Instagram). In addition, the email soporteencuestachile@gmail.com was made available to participants in case they needed more detailed information. Potential participants clicking on the invitation were directed to a screen where the objective of the study and the questionnaire were explained, followed by the request for informed consent to participate; for this they had to click the “I agree” at the bottom. The questionnaire was self-administered. Responses represent self-reported symptoms, self-reported diagnoses, and people’s perceptions, i.e., what they feel and believe they suffer from and live with, but do not provide information about diagnoses of certainty.

### 2.5 Data analysis

The sample size, calculated with 50% exposure to food risk situations and 95% confidence, was 377 individuals. The results were analyzed by descriptive statistics using SPSS and GraphPad Prism 7 software.

### 2.6 Ethical approval

The protocol was approved by the Ethics Committee of the Institute of Nutrition and Food Technology (INTA) of the University of Chile (Document #21, June 2, 2021).

## 3 Results

Of 390 individuals that answered the questionnaire, 377 provided complete data and were included in the analysis. Participants were categorized based on the diagnoses they reported. We identified 160 individuals who reported having been diagnosed with CeD, 42 NCWS, 105 individuals with FA, 36 individuals with food intolerances, and 34 individuals who reported not having been diagnosed by a healthcare professional but who experienced adverse reactions when consuming certain foods. None of the participants reported having both CeD and FA. Regarding demographic distribution, 68.4% of the participants were older than 14 years and 80.9% were female (Table 1). When asked who diagnosed their condition, most participants reported that the diagnoses were made primarily by gastroenterologists (47%) and immunologists (27.9%).

**Table 1:** Characteristics of participants by diagnosis reported at enrollment and by the specialist responsible for diagnosis.

	^a^CeD/NCWS food allergy		Food intolerance	Undiagnosed	Total
≥ 14 years	158	42	28	30	258
Females	170	70	35	30	305
Diagnosed by					
Immunologist	24	70	11	N/A^b^	105
Gastroenterologist	143	20	14	N/A	177
Pediatrician	8	3	4	N/A	15
General practitioner	20	8	5	N/A	33
Register Dietitian	2	3	1	N/A	6
Total	202	105	36	34	377

a CeD/NCWS = Celiac disease/non-celiac wheat sensitivity.

b N/A = not applicable.

Results showed that 79.8% (301) participants reported having eaten out or ordered food by delivery, of which 162 individuals (53.8%) had CeD and 77 (25.6%) FA. Frequency of these behavior was considerably variable: 24.6% did so once or twice a year, 40.2% once a month, 30.9% once a week, and only 4.3% several times a week, highlighting the complexity and diversity of food choices in this population. 33.6% of people who dined out (101 of 301) reported having experienced, at least once, an adverse reaction related to dining out. This proportion was higher among celiac (51.5%) than allergic patients (18.8%). No differences were found between eating out by diagnosis or by the other variables measured (NS).

### 3.1 Evaluation of the eating out experience

#### 3.1.1 Interaction with staff when ordering a special meal in a restaurant

**Figure 1:**
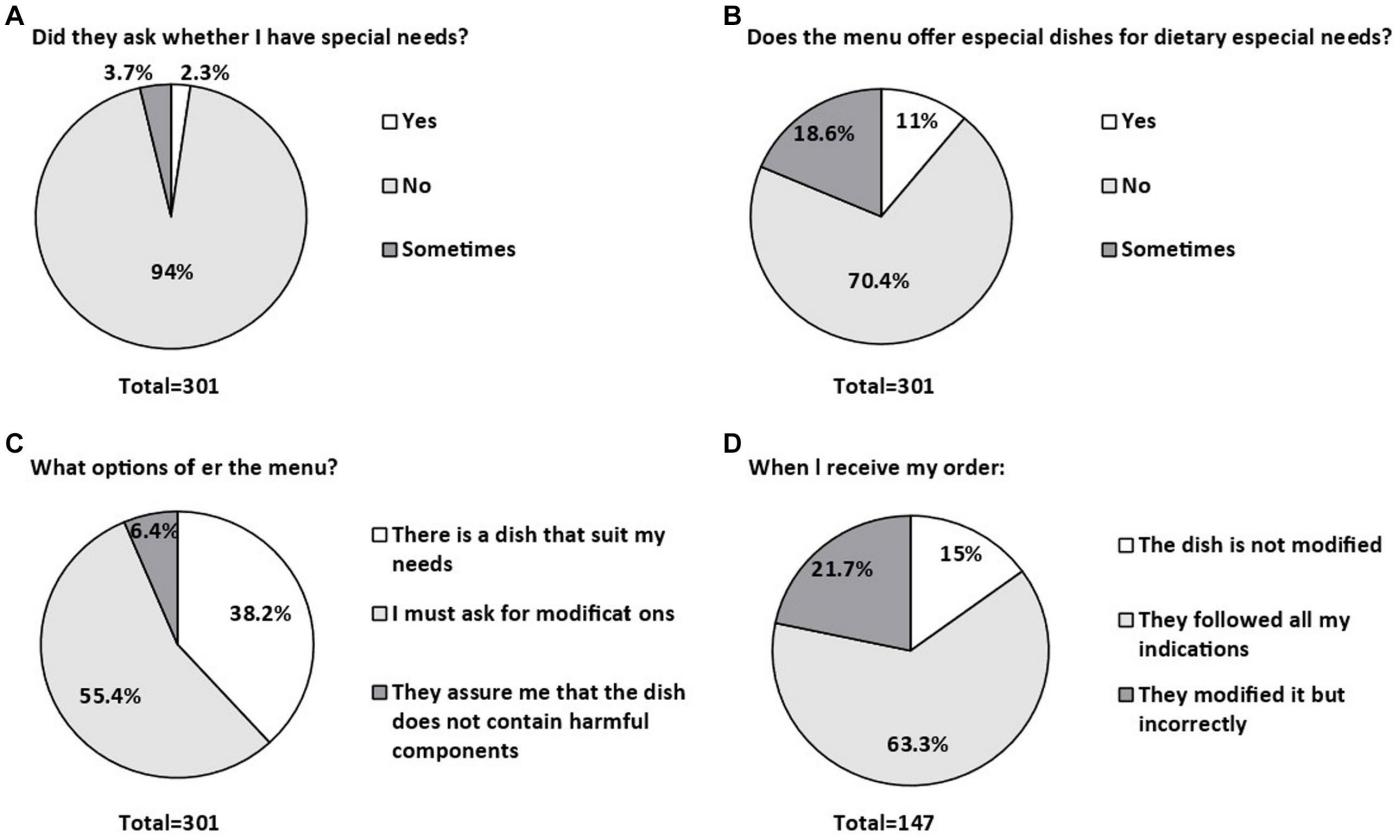
Most relevant responses related to interactions with staff when ordering a special meal at a restaurant. **(A)** asking the client for special needs. **(B)** does the menu offer special dishes for special dietary needs? **(C)** the options offered in the menu. **(D)** the dish received after ordering it.

Figure 1 shows the main responses obtained in the study. Notably, only 2.3% of waiters spontaneously asked consumers if they had any special requests regarding their food (Figure 1A). Of all restaurants reported by participants, only 11% of menus offered options labeled as gluten-free or allergen-free (Figure 1B). Of the total respondents who reported dining out (301), 149 (49.5%) persons reported that when talking with the staff they asked if they had any food allergies but no other diagnoses.

In addition, more than half of the respondents (55.4%) confirmed that the restaurant staff who served them accepted their requests to modify their dishes to accommodate their special dietary needs (Figure 1C). In 63.3% of cases, consumers confirmed that the modified dish they received was, in their opinion, suitable and safe for consumption (Figure 1D). These findings highlight the complex dynamics between consumers and food service establishments regarding special dietary demands and emphasizes the need to improve communication and understanding in this context.

Among the respondents who reported having experienced an adverse reaction while eating out, 32.3% described it as mild, moderate, or severe, without specifying how long it took for the reaction to occur; however, they did indicate the food thought to be responsible (Table 2). Self-reported severe reactions were significantly more frequent in celiac patients than in allergic patients (*p* < 0.001). No other differences were detected between adverse reactions and diagnosis, age and other variables measured.

**Table 2:** Customer’s perception of foods responsible for his/her adverse reaction and its intensity^a^.

	Severe^c^	Moderate	Mild	No reaction^c^
Gluten (%)	11.0	44.0	28.0	17.0
Peanuts (%)	3.0	3.0	13.0	81.0
Fish (%)	4.0	2.0	5.0	89.0
Seafoods (%)	5.0	5.0	6.0	84.0
Nuts (%)	3.0	3.0	12.0	82.0
Egg (%)	3.0	3.0	7.0	87.0
Cow’s milk (%)	5.0	22.0	26.0	47.0
Soy (%)	2.0	6.0	15.0	77.0
Others^b^ (%)	1.4	2.4	7.8	88.3

a Some persons reacted to more than one food.

b “Others” include reactions to sulfites, latex, monosodium glutamate, kiwi, tomato, avocado, sesame.

c Comparison of severe reactions against “all others” was significantly more frequent among celiac patients in comparison to allergic persons.

#### 3.1.2 Consumers’ perceptions regarding the knowledge of waiters or service staff

When asked about their perception of the level of knowledge and training of the people who usually serve customers in restaurants, 94.5% of respondents answered that they had no knowledge at all or that it was insufficient, and there were no differences between training and presence of adverse reactions.

#### 3.1.3 Perception of safety when eating out

When asked, “How safe do you think the food you eat when you go out to eat is?,” only 27.3% of participants felt confident about safety, while the rest felt uncertain/very uncertain.

Thorough statistical analyses explored differences between consumer characteristics and variables representing the eating out experience. With the exception of the perception of severe reactions, which was perceived more frequently by celiac patients, no differences were found by age, gender, diagnosis, frequency of eating out, and having an adverse reaction after eating food prepared away from home (NS).

## 4 Discussion

Although different clinical entities, the need for restrictive, personalized diets when eating out is a common factor among persons suffering from CeD, NCWS or FA (immune mediated conditions) and/or food intolerances (usually non-immune mediated). The results of this study show that the characteristics and consequences of eating out do not differ by the numerous variables analyzed on the part of the consumer. It is interesting that celiac patients reported significantly more severe adverse reactions after eating out (Table 2, *p* < 0.001). Unfortunately, the way this question was asked does not allow to decide whether the reaction occurred in the restaurant shortly after ingestion, or in the days following the meal, which is a relevant limitation to this study. We can only speculate that gluten may appear as a more common cause of adverse reactions among celiac persons because of its extensive use by the food industry, which makes it easier to contaminate foods and ingredients; since it is not declared in the package, it remains difficult to detect; instead, specific allergens seem easier to ask about and subsequently detect. It could also be argued that IgE-mediated allergic reactions may be more threatening, therefore patients with rapid allergies may either go out less frequently and/or participate less in this study or may be stricter in weighing the risks of accepting the final dish received. It is important that crosscontamination may be due to many different factors, like contaminated cutting board/boiling water/fryer oil/toaster/other cooking surfaces, ingredients with hidden ingredients such as dressings, sauces, herbs, and spice mixes. There are few studies assessing these aspects, an interesting one shows results that are consistent with what we found, that cross-contamination by the hands of those handling food and utensils is very common, both for gluten and for food allergens (29), even if people are not aware of it. Another interesting study reported that peanut was detectable on hands up to three hours after contact (30).

It should be emphasized that the presence of adverse reactions and their intensity as perceived by the customers were not statistically related to persons characteristics, nor they differed when comparing celiac patients with allergic persons. We interpret these results as that the characteristics of the food service, and not the person’s characteristics would be the main risk determinants when going out to eat. Several studies conducted in different countries with different cultural characteristics and dietary habits tend to show that food service characteristics and facilities are relevant factors determining the contamination risk (31–33). It is therefore important to understand that the responsibility for adverse reactions is shared between consumers and food providers. Our results show that consumers in general explain the need for his/her special diet because he/she feels it is part of his commitment to treatment; some authors have interpreted this as that it is the consumer’s responsibility to handle the situation of eating out correctly (34). However, food service providers should share responsibility. In recent years, significant gaps have been reported in the information provided by food services about gluten-free food availability on menus and other documents (35–38).

Studies show that allergic patients prefer to rely on written information, as this avoids the need to discuss the problem at the time the staff is serving them (24). People with CeD also rely on written information when purchasing or ordering food, and their social frustration and isolation experienced when dining does not differ from that reported by people with allergies (39–41). Customers with FA are often reported to be reluctant to share personal relevant information, possibly for fear of being seen as “picky” or “troublesome” customers (42, 43). However, verbal communication is critical when food is prepared in a manner different from that used by the person at home (44). In the United States (45), a study of restaurant service staff found that including a statement on the menu advising customers to inform restaurant staff of their FAs was perceived as one of the most effective communication strategies for preventing potential allergic reactions to food in restaurants.

While lifelong strict gluten-free diet is the only treatment for CeD, strict avoidance of causal allergens is the primary strategy to prevent FA (24, 25). Today, additional groups follow the gluten-free diet because they feel that this diet is “healthy” or helps losing weight (31). This complicates the assessments because their requirements are different and, being an option and not a treatment, they have no consequences after gluten/allergen ingestion. We could speculate that at least some of the people who stated in our survey that they had food intolerance without a diagnosis and that they followed the gluten-free diet “to see if it was good for my health” belong to this group. Although confusing, these numerous groups are driving the market to offer gluten-free menus in restaurants and food services, even when these may lack the capacity and expertise to produce safe gluten-free or allergen-free foods (32). In the case of the gluten-free diet, a common mistake is to assume that eliminating wheat, barley, rye and contaminated oats as an ingredient results in a safe gluten-free dish, ignoring the role of additives and cross-contamination (33).

This study confirms that eating away from home is now a common practice in persons following restrictive diets (100% of respondents reported eating some food not prepared at home), and it should be noted that most respondents reported difficulty finding information about the exact contents of foods and dishes served in restaurants. Consumer perceptions indicate that gluten, peanuts, and eggs were the most common causes of adverse reactions when consumed away from home or through delivery. Unfortunately, due to the methodology used, it was not possible to further analyze the role of specific allergens.

The perception of food safety is particularly relevant, being mostly of insecurity, consumers feel that the personnel in the collective food services have little knowledge about food intolerances, FA and CeD. Providing the knowledge needed to properly manage consumers’ dietary needs depends on the owner, manager, or person in charge of the food service. Staff personnel typically receives basic training on the hygienic requirements for handling food, but little attention is paid to issues such as special diets, CeD, or FA. In fact, the evidence obtained in this study shows that even when restaurant staff have received some training, they do not feel confident enough to know for sure that what they are serving to a special customer is safe. Customers with FA, CeD, and other special dietary needs feel that they must be self-reliant and vigilant in ensuring their own safety (42, 46).

Another relevant factor in the adverse situation described is the lack of or inadequacy of regulations and monitoring of standards, depending on local health authorities. Priorities are set, but the state government often leaves food/diet related issues in the low priority levels. This means that the chances of improvement depend on the good will of food services; although it is true that their practices remain voluntary, they should improve them and keep their staff informed and trained in these aspects. The extra effort that food services would have to invest to make their food safe would be of enormous benefit to people with all types of food-intolerance (47–49). Since a significant proportion of the population is now concerned about their diet and willing to change some of their habits in order to eat healthier, the refinement of the range and safety of the food offered would improve the image that customers have of food providers.

It is worth commenting some limitations of this study. Self-reported data have methodological limitations yet, they provide valuable insight into consumers’ experiences, the way they live and face their health problems. (1) Variability of reactions: Reactions to food in each of the conditions approached can vary greatly in duration and type of symptoms. Deciding how to record data in a study like this is challenging because each person may have a unique way to perceive his/her condition; (2) Wide range of responses: In CeD and NCWS there is a wide range of responses, from clinically obvious presentations to less apparent extraintestinal or asymptomatic forms of these diseases. Symptoms may overlap with other gastrointestinal conditions like irritable bowel syndrome (IBS), making difficult to attribute symptoms to a single dietary component. This is especially true when the methodology favors achieving a larger sample size, as in this study, weakening the possibility of obtaining firm diagnoses; (3) Lack of clear definitions: With the exception of CeD, NCWS, and WA, the term “food intolerance” lacks of a consensus definition and includes a variety of conditions, making difficult to accurately identify individuals suitable for studies such as this one; and (4) Self-diagnosis bias: In many cases, participants rely on their own perception and experience to determine whether they have an allergy, sensitivity, or intolerance. This can lead to over- or under estimations of prevalence of these conditions. In addition, there are other type of intervening factors to be considered, as food poisoning or consumption of large portions of food that usually are not controlled. Symptoms in irritable bowel syndrome (IBS) and celiac individuals on a gluten-free diet may overlap, introducing another uncertainty factor when interpreting self-reports. In contrast, FA symptoms are often more objective reactions, reducing uncertainty when compared to CeD. These differences may lead to overestimating adverse responses in CeD and NCWS, while the association of IBS with FA may underscore the importance of symptoms. These considerations emphasize the intricate nature of gluten-related conditions and support the need of individualized approaches when managing restrictive diets (50).

We conclude that people with food intolerances of different origins and special dietary needs are at risk when eating out; our results indicate that the current offer of restaurants and food services does not meet the food safety requirements that people with specific restrictive diets have, especially allergic and celiac persons. Communication between food service personnel and consumers needs to be improved, and personnel needs to be trained. Although informed consumers and properly trained personnel seem crucial, this is insufficient without the participation of food service providers. They should recognize that the consumer’s characteristics are not significant factors in determining a safe going out to eat; rather, food safety appears associated with restaurant or food service characteristics, and these would be what makes the experience unsafe and unsatisfactory. The need for properly monitored regulations to supervise safety of gluten-free and allergen-free foods is urgent.

## Data availability statement

The raw data supporting the conclusions of this article will be made available by the authors, without undue reservation.

## Ethics statement

The studies involving humans were approved by Ethics Committee of the National Institute of Agricultural Research (INTA) of the University of Chile (Document #21, June 2, 2021). The studies were conducted in accordance with the local legislation and institutional requirements. The participants provided their written informed consent to participate in this study.

## Author contributions

XF-G: Conceptualization, Formal analysis, Investigation, Project administration, Visualization, Writing – original draft. MO-L: Supervision, Visualization, Writing – original draft. JR: Conceptualization, Data curation, Formal analysis, Methodology, Writing – review & editing. MP: Data curation, Formal analysis, Visualization, Writing – original draft. HL: Conceptualization, Supervision, Writing – original draft. MA: Conceptualization, Formal analysis, Methodology, Supervision, Writing – review & editing.

## Footnotes

^1^www.inta.uchile.cl

^2^www.coacel.cl

^3^www.creciendoconalergias.cl

## References

[1] Allergy UK. Food-allergy. Allergy UK. (2023) (Accessed June 5, 2023]. Available at: https://www.allergyuk.org/types-of-allergies/food-allergy/

[2] Xu YS Kastner M Harada L Xu A Salter J Waserman S. Anaphylaxis-related deaths in Ontario: a retrospective review of cases from 1986 to 2011. Allergy Asthma Clin Immunol. (2014) 10:38. doi: 10.1186/1710-149210-38 25670935 PMC4322510

[3] Sapone A Bai JC Ciacci C Dolinsek J Green PHR Hadjivassiliou M. Spectrum of gluten-related disorders: consensus on new nomenclature and classification. BMC Med. (2012) 10:13. doi: 10.1186/1741-7015-10-13, PMID: 22313950 PMC3292448

[4] Barada K Abu Daya H Rostami K Catassi C. Celiac disease in the developing world. Gastrointest Endosc Clin N Am. (2012) 22:773–96. doi: 10.1016/j.giec.2012.07.002 23083993

[5] Food Allergy Research & Education (FARE). Food allergy facts & statistics for the U. S. (2019). Available at: https://www.foodallergy.org/file/facts-stats.pdf

[6] Pawankar R. Allergic diseases: a global public health issue. Asian Pac J Allergy Immunol. (2012) 30:39–41. doi: 10.1097/ACI.0b013e32834ec13b

[7] Radlović N Leković Z Radlović V Simić D Ristić D Vuletić B. Food allergy in children. Srp Arh Celok Lek. (2016) 144:99–103. doi: 10.2298/SARH1602099R 27276868

[8] Ebisawa M Ito K Fujisawa T. Japanese guidelines for food allergy 2017. Allergol Int. (2017) 66:248–64. doi: 10.1016/j.alit.2017.02.001, PMID: 28285847

[9] Arasi S Mennini M Valluzzi R Riccardi C Fiocchi A. Precision medicine in food allergy. Curr Opin Allergy Clin Immunol. (2018) 18:438–43. doi: 10.1097/ACI.0000000000000465 30015641

[10] Green PHR Lebwohl B Greywoode R. Celiac disease. J Allergy Clin Immunol. (2015) 135:1099–106. doi: 10.1016/j.jaci.2015.01.044 25956012

[11] Taylor AK Lebwohl B Snyder CL Green PHR In: Adam MP Mirzaa GM Pagon RA Wallace SE LJH B Gripp KW, editors. Seattle (WA): University of Washington, Seattle. Celiac disease (1993). 20301720

[12] Lionetti E Pulvirenti A Vallorani M Catassi G Verma AK Gatti S. Re-challenge studies in non-celiac gluten sensitivity: a systematic review and meta-analysis. Front Physiol. (2017) 8:621. doi: 10.3389/fphys.2017.00621, PMID: 28928668 PMC5591881

[13] Gupta RS Warren CM Smith BM Jiang J Blumenstock JA Davis MM. Prevalence and severity of food allergies among US adults. JAMA Netw Open. (2019) 2:e185630–08. doi: 10.1001/jamanetworkopen.2018.5630, PMID: 30646188 PMC6324316

[14] Sicherer SH Sampson HA. Food allergy: a review and update on epidemiology, pathogenesis, diagnosis, prevention, and management. J Allergy Clin Immunol. (2018) 141:41–58. doi: 10.1016/j.jaci.2017.11.003, PMID: 29157945

[15] Food and Drug Administration (FDA). Food Allergies. (2023) (Accessed 2023 Jun 11). Available at: https://www.fda.gov/food/food-labeling-nutrition/foodallergies#:$~$:text=This

[16] Bock SA Muoz-Furlong A Sampson HA. Fatalities due to anaphylactic reactions to foods. J Allergy Clin Immunol. (2001) 107:191–3. doi: 10.1067/mai.2001.112031 11150011

[17] Zugasti MA. Intolerancia alimentaria. Endocrinol y Nutr. (2009) 56:241–50. doi: 10.1016/S1575-0922(09)71407-X, PMID: 19627745

[18] Lomer MCE Parkes GC Sanderson JD. Review article: lactose intolerance in clinical practice—myths and realities. Aliment Pharmacol Ther. (2008) 27:93–103. doi: 10.1111/j.1365-2036.2007.03557.x, PMID: 17956597

[19] Food Allergy Research & Education (FARE). Facts and statistics. (2023) (Accessed June 5, 2023]. Available at: https://www.foodallergy.org/resources/facts-andstatistics

[20] Lefèvre S Abitan L Goetz C Frey M Ott M de Blay F. Multicenter survey of restaurant staff’s knowledge of food allergy in eastern France. Allergo J Int. (2019) 28:57–62. doi: 10.1007/s40629-018-0062-2

[21] Administration F, Drug. Food allergen labeling and consumer protection. (2004). Available at: https://www.fda.gov/food/food-allergensgluten-free-guidancedocuments-regulatory-information/food-allergen-labeling-and-consumer-protectionact-2004-falcpa

[22] UE. Reglamento UE N.^o^ 1169/2011. (2011).

[23] FAO/WHO. Codex general standard for food additives. (1995). Available at: https://www.fao.org/fao-who-codexalimentarius/codex-texts/dbs/gsfa/en/

[24] Barnett J Begen FM Gowland MH Lucas JS. Comparing the eating out experiences of consumers seeking to avoid different food allergens. BMC Public Health. (2018) 18:1263. doi: 10.1186/s12889-018-6117-y 30442121 PMC6238278

[25] Taylor SL Baumert JL. Cross-contamination of foods and implications for food allergic patients. Curr Allergy Asthma Rep. (2010) 10:265–70. doi: 10.1007/s11882-010-0112-4 20425003

[26] Weiss CC Muñoz-Furlong A. Fatal food allergy reactions in restaurants and food -service establishments: strategies for prevention. Food Prot Trends. (2008)

[27] Dubé C Rostom A Sy R Cranney A Saloojee N Garritty C. The prevalence of celiac disease in average-risk and at-risk Western European populations: a systematic review. Gastroenterology. (2005) 128:S57–67. doi: 10.1053/j.gastro.2005.02.014 15825128

[28] Villanueva M Oyarzún A Leyton B González M Navarro E Canales P. Changes in age at diagnosis and nutritional course of celiac disease in the last two decades. Nutrients. (2020) 12:156. doi: 10.3390/nu12010156, PMID: 31935859 PMC7019995

[29] Verrill L Zhang Y Kane R. Food label usage and reported difficulty with following a gluten-free diet among individuals in the USA with coeliac disease and those with noncoeliac gluten sensitivity. J Hum Nutr Diet. (2013) 26:479–87. doi: 10.1111/jhn.12032, PMID: 23347179

[30] Brough HA Makinson K Penagos M Maleki SJ Cheng H Douiri A. Distribution of peanut protein in the home environment. J Allergy Clin Immunol. (2013) 132:623–9. doi: 10.1016/j.jaci.2013.02.035 23608728

[31] Arslain K Gustafson CR Baishya P Rose DJ. Determinants of gluten-free diet adoption among individuals without celiac disease or non-celiac gluten sensitivity. Appetite. (2021) 156:104958. doi: 10.1016/j.appet.2020.104958 32919023

[32] Kim YH Duncan J Chung BW. Involvement, satisfaction, perceived value, and revisit intention: a case study of a food festival. J Culin Sci Technol. (2015) 13:133–58. doi: 10.1080/15428052.2014.952482

[33] Nwaru BI Muraro A Sheikh A. Charting a research agenda for understanding the epidemiology of food allergy in adults in Europe. Allergy. (2014) 69:975–7. doi: 10.1111/all.12434, PMID: 25041524

[34] Kwon J Lee YM Wen H. Knowledge, attitudes, and behaviors about dining out with food allergies: a cross-sectional survey of restaurant customers in the United States. Food Control. (2020) 107:106776. doi: 10.1016/j.foodcont.2019.106776

[35] Marsilio I Savarino EV Barberio B Lorenzon G Maniero D Cingolani L. A survey on nutritional knowledge in coeliac disease compared to inflammatory bowel diseases patients and healthy subjects. Nutrients. (2020) 12:1110. doi: 10.3390/nu12041110, PMID: 32316215 PMC7230195

[36] Dupuis R Kinsey EW Spergel JM Brown-Whitehorn T Graves A Samuelson K. Food allergy Management at School. J Sch Health. (2020) 90:395–406. doi: 10.1111/josh.12885 32124441

[37] Schellpfeffer NR Leo HL Ambrose M Hashikawa AN. Camp leadership perspectives on food allergy-related anaphylaxis events and training for camp staff: a National Survey of summer camps. J Allergy Clin Immunol Pract. (2020) 8:1247–1252.e1. doi: 10.1016/j.jaip.2019.11.014, PMID: 31770654

[38] Tsuang A Demain H Patrick K Pistiner M Wang J. Epinephrine use and training in schools for food-induced anaphylaxis among non-nursing staff. J Allergy Clin Immunol Pract. (2017) 5:1418–1420.e3. doi: 10.1016/j.jaip.2017.04.014, PMID: 28506422

[39] Muraro A Hoffmann-Sommergruber K Holzhauser T Poulsen LK Gowland MH Akdis CA. EAACI food allergy and anaphylaxis guidelines. Protecting consumers with food allergies: understanding food consumption, meeting regulations and identifying unmet needs. Allergy. (2014) 69:1464–72. doi: 10.1111/all.12453 24888964

[40] Zingone F Siniscalchi M Carpinelli L Iovino P Zingone L Ciacci C. The celiac disease patients’ ability to experience pleasure. Gastroenterol Res Pract. (2019) 2019:1–5. doi: 10.1155/2019/2030751 PMC6421785 30944557

[41] Bioletti L Ferrero BM Magistroni P Teriaca MJ Ropolo S Emma L. Gluten-free meals in public catering. Ann Ig. (2021) 33:3–9. doi: 10.7416/ai.2021.2403, PMID: 33354691

[42] Kwon J Lee YM. Exploration of past experiences, attitudes and preventive behaviors of consumers with food allergies about dining out: a focus group study. Food Prot Trends. (2012) 32:736–46.

[43] Barnett J Leftwich J Muncer K Grimshaw K Shepherd R Raats MM. How do peanut and nut-allergic consumers use information on the packaging to avoid allergens? Allergy. (2011) 66:969–78. doi: 10.1111/j.1398-9995.2011.02563.x, PMID: 21320134

[44] Begen FM Barnett J Payne R Roy D Gowland MH Lucas JS. Consumer preferences for written and oral information about allergens when eating out. PLoS One. (2016) 11:1–12. doi: 10.1371/journal.pone.0156073 PMC4880205 27223698

[45] Wen H Lee Y. Effects of message framing on food allergy communication: a crosssectional study of restaurant customers with food allergies. Int J Hosp Manag. (2019) 89:102401. doi: 10.1016/j.ijhm.2019.102401

[46] Boyce JA Assa’ad A Burks AW Jones SM Sampson HA Wood RA. Guidelines for the diagnosis and Management of Food Allergy in the United States: summary of the NIAID-sponsored expert panel report. Nutr Res. (2011) 31:61–75. doi: 10.1016/j.nutres.2011.01.001, PMID: 21310308 PMC4249938

[47] Abbot JM Byrd-Bredbenner C Grasso D. “Know before you serve”: developing a food-allergy fact sheet. Cornell Hotel Restaur Adm Q. (2007) 48:274–83. doi: 10.1177/0010880407302779

[48] Ahuja R Sicherer SH. Foodallergy management from the perspective of restaurant and food establishment personnel. Ann Allergy Asthma Immunol. (2007) 98:344–8. doi: 10.1016/S1081-1206(10)60880-0, PMID: 17458430

[49] Kronenberg SA. Food allergy risk management: more customers, less liability. J Foodserv Bus Res. (2012) 15:117–21. doi: 10.1080/15378020.2012.652017

[50] Mearin ML Agardh D Antunes H Al-Toma A Auricchio R Castillejo G. ESPGHAN position paper on management and follow-up of children and adolescents with celiac disease. J Pediatr Gastroenterol Nutr. (2022) 75:369–86. doi: 10.1097/MPG.0000000000003540, PMID: 35758521

